# Association between different sodium–glucose co-transporter 2 inhibitors and the risk of dementia: a network meta-analysis

**DOI:** 10.3389/fendo.2026.1777151

**Published:** 2026-08-04

**Authors:** Zhongru Meng, Hui Wu, Yang Miao, Wang Qi, Mingzhu Sha

**Affiliations:** Department of Pharmacy, Yancheng First Hospital Affiliated to Nanjing University Medical School - Yancheng First People’s Hospital, Yancheng, China

**Keywords:** cognitive impairment, dementia, network meta-analysis, sodium-glucose cotransporter 2 inhibitors, systematic review

## Abstract

**Background:**

Sodium-glucose cotransporter 2 inhibitors (SGLT2i), as key therapeutic agents for type 2 diabetes, have in recent years been recognized as potentially exerting neuroprotective effects on the central nervous system. However, systematic comparative evidence remains lacking regarding whether different SGLT2i exhibit differential effects on dementia risk. By conducting a network meta-analysis to compare the association between different SGLT2i and the risk of dementia onset, the relative efficacy of each drug in reducing dementia risk is determined.

**Methods:**

A systematic search was conducted across databases including PubMed, Embase, Web of Science, and the Cochrane Library, from their inception to the search cutoff date of 1 December 2025. Randomized controlled trials and observational studies comparing SGLT2i with other antidiabetic medications or placebo, and reporting dementia outcomes, were included. A network meta-analysis employing a random-effects model was conducted. Pooled effect sizes were expressed as hazard ratios with 95% confidence intervals. Relative efficacy among different drugs was ranked using the probability of ranking, whilst heterogeneity and consistency were assessed.

**Results:**

Six cohort studies involving 845,433 patients were included in this analysis. The results of the network meta-analysis indicated that canagliflozin (HR = 0.75, 95% CrI: 0.59, 0.98), Dapagliflozin (HR = 0.75, 95% CrI: 0.59, 0.98), and Empagliflozin (HR = 0.69, 95% CrI: 0.55, 0.85) significantly reduced the risk of dementia onset. Empagliflozin had the highest probability of being ranked as the most effective treatment (81.2%). However, no statistically significant differences were observed between empagliflozin and dapagliflozin or canagliflozin in the network comparisons. For Alzheimer’s disease, Dapagliflozin (HR = 0.68, 95% CrI: 0.48, 0.96) and Empagliflozin (HR = 0.62, 95% CrI: 0.45, 0.87) also demonstrated significant risk reduction, whilst direct comparisons between Empagliflozin and other agents showed no significant differences. For vascular dementia, Empagliflozin (HR = 0.63, 95% CrI: 0.46, 0.87) and Canagliflozin (HR = 0.71, 95% CrI: 0.48, 0.98) also demonstrated favorable outcomes.

**Conclusions:**

The findings of this study indicate that dapagliflozin, empagliflozin, and canagliflozin, particularly empagliflozin, demonstrate significant potential in reducing the incidence of dementia and related cognitive impairments. Compared with DPP-4 inhibitors, these agents effectively lower the risk of dementia, Alzheimer’s disease, and vascular dementia, offering an effective therapeutic option for diabetic patients, particularly the elderly.

## Background

Dementia is a major public health challenge, and its global burden is expected to increase substantially with population ageing (1–3). Patients with type 2 diabetes have a significantly higher risk of developing dementia than the general population, possibly through mechanisms including hyperglycemia, insulin resistance, chronic inflammation, oxidative stress, and vascular dysfunction (4, 5). Therefore, identifying antidiabetic therapies with potential neuroprotective effects has become an important research priority (6).

Sodium-glucose cotransporter 2 inhibitors (SGLT2is) are widely used glucose-lowering agents that also provide cardiovascular and renal benefits (7, 8). Emerging experimental and observational evidence suggests that SGLT2is may reduce dementia risk through multiple mechanisms, including improving insulin sensitivity, attenuating inflammation and oxidative stress, and enhancing cerebral perfusion (9, 10). Beyond cardiovascular and renal benefits, the potential effects of SGLT2i on the central nervous system are increasingly attracting attention. Basic research suggests these agents may exert neuroprotective effects through multiple pathways, including enhancing insulin sensitivity, reducing inflammatory responses, mitigating oxidative stress, improving cerebral blood perfusion, and regulating energy metabolism (11, 12). Observational studies have indicated that diabetic patients using SGLT2i exhibit a lower risk of dementia compared to those on other classes of antidiabetic medications. However, conclusions from existing studies are not entirely consistent due to variations in research design, study populations, and comparator drugs (13).

It is noteworthy that SGLT2i are not a homogeneous class of drugs; specific agents differ in pharmacokinetic characteristics, selectivity, half-life, and potential non-antidiabetic effects (14). Consequently, whether different SGLT2i exert identical or divergent effects on dementia risk remains an unresolved question. Previous conventional pairwise meta-analyses (15, 16) primarily compared SGLT2 inhibitors as a drug class or compared individual SGLT2 inhibitors with DPP-4 inhibitors but could not directly compare the relative effects of different SGLT2 inhibitors because head-to-head studies are lacking. Network meta-analysis overcomes this limitation by integrating both direct and indirect evidence, allowing simultaneous comparison and ranking of individual SGLT2 inhibitors within a single analytical framework.

Consequently, this study employs a network meta-analysis to compare the association between different SGLT2i and dementia risk, clarifying the relative efficacy of each drug in potential cognitive protection. This provides scientific evidence for individualized medication selection in diabetic patients and the early prevention of dementia.

## Methods

This systematic review and network meta-analysis was conducted in accordance with the PRISMA 2020 statement (17) and was prospectively registered in PROSPERO (CRD420251252692).

### Literature retrieval

Two researchers independently conducted literature searches, systematically retrieving studies from PubMed, Embase, Cochrane Library, Web of Science from each database’s inception to 1 December. A combined strategy of subject headings and free-text terms was employed. Key English search terms included “Sodium-Glucose Transporter 2 Inhibitors,” “Dementia,” “Alzheimer Syndrome,” and “Dementias.” To prevent omission of relevant studies, a retrospective search of references from included publications was also conducted. No language restrictions were applied during the search. The specific search strategy is detailed in **Supplementary Material** **Supplementary Table 1**.

### Inclusion and exclusion criteria

#### Inclusion criteria

Subjects were adults aged ≥18 years with a confirmed diagnosis of type 2 diabetes mellitus and no baseline dementia or cognitive impairment.

Interventions comprised treatment with sodium-glucose cotransporter 2 inhibitors, including dapagliflozin, empagliflozin, and canagliflozin.

The control intervention comprises treatment with dipeptidyl peptidase-4 inhibitors.

Outcome measures: Dementia outcome ascertainment was generally consistent across the included studies. Most studies identified dementia using validated diagnostic algorithms based on ICD-9/ICD-10, Read, or SNOMED diagnostic codes extracted from nationwide electronic health records or administrative healthcare databases. Several studies further incorporated antidementia medication prescriptions to improve diagnostic accuracy. Dementia was evaluated as a predefined outcome in all included cohort studies, with Alzheimer’s disease and vascular dementia additionally reported as secondary outcomes in some studies (**Table 1**).

**Table 1:** SUCRA rank results.

Study	Outcome definition	Data source	Diagnostic method	Prespecified outcome
Abdullah 2025	Incident dementia	CPRD Aurum	Read/SNOMED codes + antidementia medications	Primary outcome
Chiang 2025	Incident dementia	TriNetX	ICD-10 codes	Primary outcome
Hong 2024	Incident dementia	NHIS	ICD-10 codes + antidementia medications	Primary outcome
Liu 2025	Incident dementia	TriNetX	ICD-10 codes	Primary outcome
Tang 2025	ADRD	OneFlorida+	ICD-9/10 (CCW algorithm)	Primary outcome
Wu 2023	Incident dementia	Ontario administrative database	Validated administrative algorithm	Primary outcome

Study designs include randomized controlled trials, cohort studies, or case-control studies, with data available for effect size calculation.

#### Exclusion criteria

Studies including participants diagnosed with dementia or cognitive impairment at baseline, or involving children, adolescents, or non-type 2 diabetes patients.

Studies failing to clearly distinguish between sodium-glucose cotransporter 2 inhibitors and dipeptidyl peptidase-4 inhibitor (DDP4i) exposure groups, or with significant gaps in medication information.

Studies failing to report dementia incidence, where effect sizes could not be extracted or calculated, or those assessing only short-term cognitive function without dementia diagnosis.

Reviews, systematic reviews, meta-analyses, conference abstracts, case reports, case series, and animal or basic research.

Studies with duplicated data, critical data severely missing and inaccessible, or those with very high risk of bias significantly compromising result reliability.

### Data extraction

Two researchers independently extracted data from studies meeting inclusion criteria. Disagreements were resolved through discussion or adjudication by a third researcher. Extracted information comprised the first author’s name, publication year, country or region of study, study design, sample size, mean age of participants, and gender composition. Regarding interventions, the specific drug names of sodium-glucose cotransporter 2 inhibitors were recorded. For outcome measures, the following were extracted: type of dementia (dementia, Alzheimer’s disease, or vascular dementia); diagnostic criteria or basis for determination; reported effect size (hazard ratio) with its 95% confidence interval. Where multiple effect sizes were reported, results adjusted for multiple variables were prioritized. For studies with incomplete data or missing key information, attempts were made to contact original authors to obtain supplementary data.

### Risk of bias

The included cohort studies underwent methodological quality assessment using the Newcastle–Ottawa Scale (18). This scale evaluates study quality across three domains: participant selection, comparability of groups, and outcome assessment, with a maximum score of 9 points. The participant selection domain primarily assesses the representativeness of exposed versus unexposed groups, the method of exposure factor determination, and the absence of outcome occurrence at baseline; The comparability of groups section primarily assessed whether important confounding factors were controlled for; the outcome assessment section mainly examined the method of outcome determination, the adequacy of follow-up duration, and the occurrence of loss to follow-up. Scoring was performed independently by two researchers. In the event of disagreement, resolution was achieved through discussion or adjudication by a third researcher. Studies scoring ≥7 points on the Newcastle–Ottawa Scale were generally considered to be of high quality.

### Data analysis

In R 4.5.1 software, Bayesian network meta-analysis was conducted using the gemtc package. All analyses employed random-effects models for network meta-analysis, with HR and their 95% confidence intervals serving as effect measures to evaluate the association between various sodium-glucose cotransporter 2 inhibitors and the risk of dementia. Model parameters employed non-informative prior distributions: the overall mean of treatment effects followed a normal distribution with mean 0 and variance 10,000; the standard deviation of between-studies heterogeneity parameters followed either a half-normal or uniform distribution (range 0–2). Model parameters were estimated using Markov chain Monte Carlo methods, employing four independent chains, each run for 50,000 iterations. The first 20,000 iterations were discarded as the burn-in period, with the remaining iterations used for posterior inference (19). Thinning intervals of 10 were applied to reduce autocorrelation. Global consistency of the evidence network was assessed using the global consistency model, with local inconsistencies tested via node splitting. Heterogeneity was assessed via the variance parameter in the random-effects model. Probability-weighted rankings for each intervention were calculated from the posterior distribution, with area under the cumulative ranking curve further computed to evaluate the relative ranking of different sodium-glucose cotransporter 2 inhibitors in reducing dementia risk. Network and funnel plots were generated using Stata 15 software.

## Results

### Literature screening results

**Figure 1:**
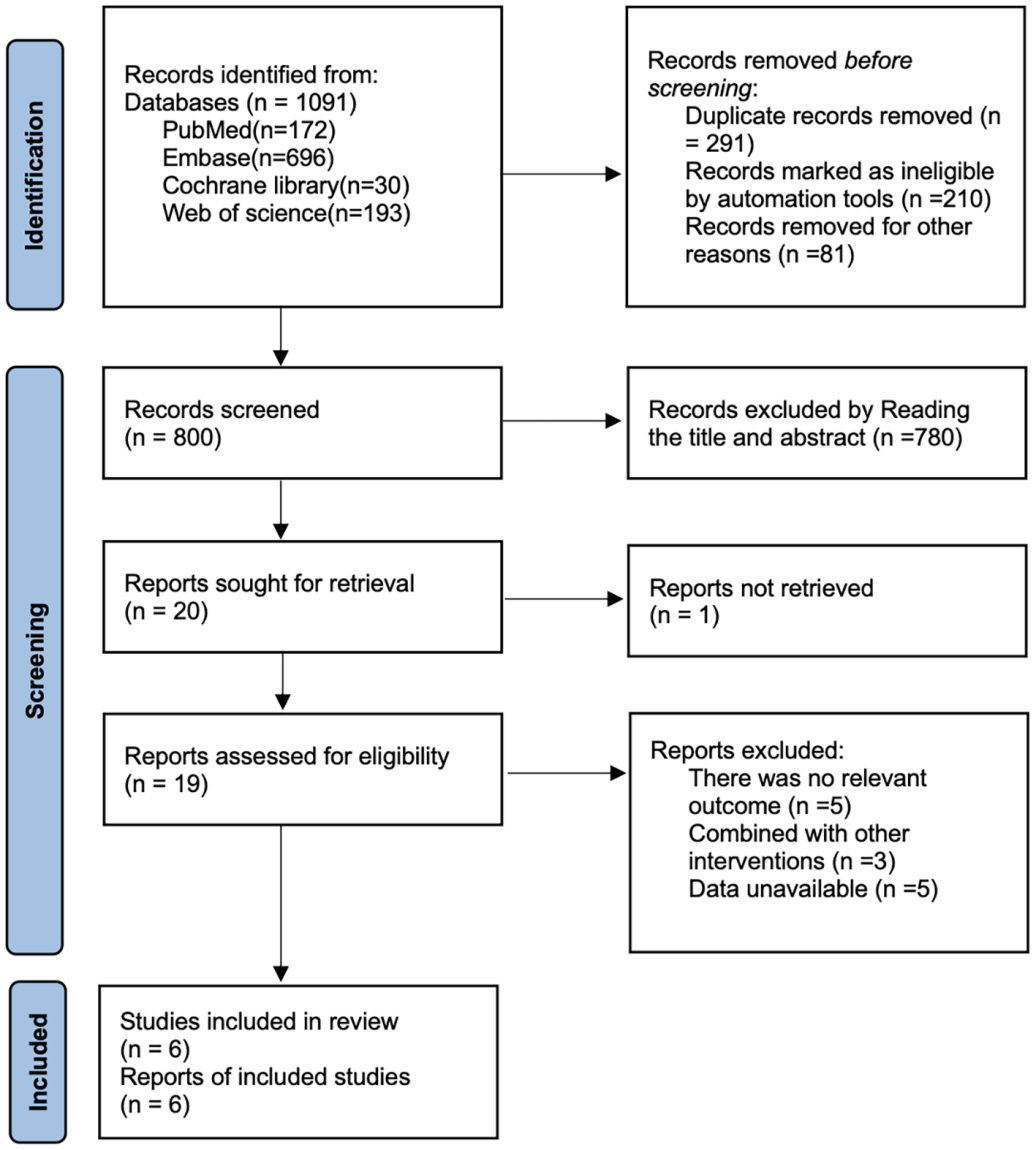
Flowchart of literature retrieval.

As shown in **Figure 1**, a total of 1,091 articles were retrieved through searches of PubMed (n=172), Embase (n=696), the Cochrane Library (n=30), and Web of Science (n=193). After removing 291 duplicate records, 780 articles were excluded based on title and abstract screening, and a further 13 were excluded following full-text review. Ultimately, 6 cohort studies (20–25) were included.

### Basic characteristics of included study

The studies included in this review are presented in **Table 2**. A total of six studies involving 845,433 patients were included, published between 2024 and 2025. Two articles originated from China, two from Canada, one from South Korea, and one from the United States. The average age was 65.74 years. The drugs involved were Dapagliflozin; Empagliflozin; and Canagliflozin. All articles mentioned dementia, with two specifically addressing vascular dementia and Alzheimer’s disease.

**Table 2:** Table of basic characteristics of literature.

Author	Year	Country	Study design	Sample size	Gender (M/F)	Mean age(years)	Type of SGLT-2i	Outcome
Abdullah	2025	Canada	cohort study	118006	78006/40000	61.8	Dapagliflozin; Empagliflozin; Canagliflozin	Dementia
Chiang	2025	China	cohort study	31802	14923/16879	66.25	Canagliflozin; Dapagliflozin; Empagliflozin;	Dementia; vascular dementia; Alzheimer’s disease;
Hong	2024	South Korea	cohort study	427630	17712/250518	68	Dapagliflozin; Empagliflozin;	Dementia
Liu	2025	China	cohort study	160752	68946/76274	69.6	Canagliflozin; Dapagliflozin; Empagliflozin;	Dementia; vascular dementia; Alzheimer’s disease
Tang	2025	USA	cohort study	34185	16684/17321	65.2	Canagliflozin; Dapagliflozin; Empagliflozin;	Dementia
Wu	2025	Canada	cohort study	73058	44657/28041	72.4	Canagliflozin; Dapagliflozin; Empagliflozin;	Dementia

### Risk bias results

This study employed the NOS scoring system. The results (**Supplementary Material** **Supplementary Table 2**) indicate that three of the included studies scored 9 points, while three scored 8 points. The primary deductions stemmed from baseline differences observed in some studies. Nevertheless, all studies included in this review were of high quality.

### Results of consistency modeling

The current study used a random-effects model to compare the difference in DIC between consistent and inconsistent modeling, and the absolute value of the difference in DIC was <5, The results (**Supplementary Material** **Supplementary Table 3**) indicate that dementia; Alzheimer’s disease and Vascular dementia have consistency. Model convergence was assessed using the Gelman–Rubin diagnostic, and all potential scale reduction factors (Rhat) were close to 1.0, indicating satisfactory convergence (**Supplementary Material** **Supplementary Figure 4**).

### Dementia

**Figure 2:**
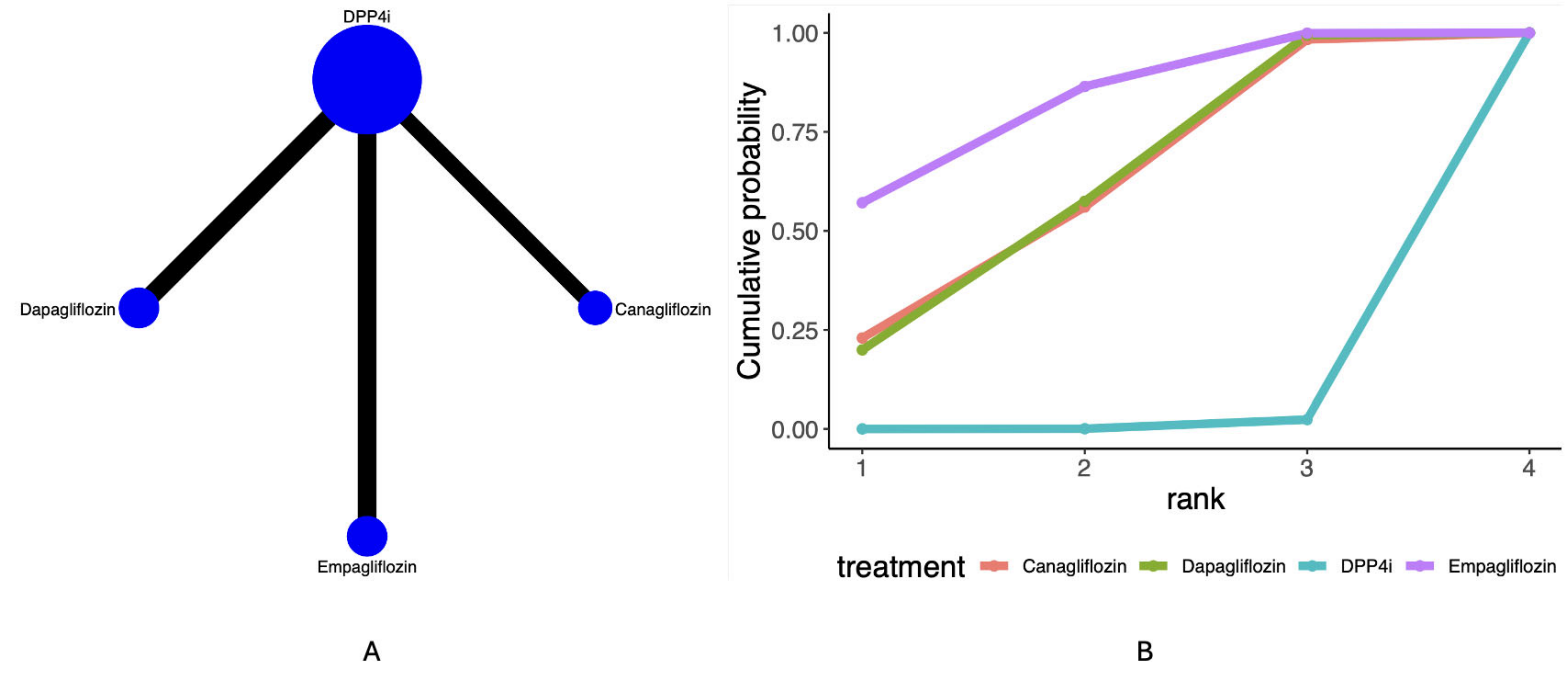
Results of network meta-analysis of dementia **(A)** network plot; **(B)** cumulative probability plot).

All 6 studies mentioned dementia. The network of analyses diagram (**Figure 2A**) indicates direct comparisons between dapagliflozin, empagliflozin, and canagliflozin versus DDP4 inhibitors. The league table (**Supplementary Material** **Supplementary Table 4**) suggests that compared with DDP4 inhibitors, canagliflozin [HR = 0.75, 95%CrI (0.59, 0.98)], dapagliflozin [HR = 0.75, 95%CrI (0.59, 0.98)], and empagliflozin [HR = 0.69, 95%CrI (0.55, 0.85)] demonstrated reduced dementia incidence compared to DDP4i. However, empagliflozin showed no statistically significant difference compared to dapagliflozin [HR = 0.92, 95%CrI (0.67, 1.24)] or canagliflozin [HR = 0.92, 95%CrI (0.65, 1.26)]. Cumulative probability curve ranking (**Figure 2B**; **Table 3**) indicated empagliflozin (81.2%) > canagliflozin (59.1%) > dapagliflozin (58.9%).

**Table 3:** Sucra rank results.

Outcomes	Dementia (%)	Alzheimer’s disease (%)	Vascular dementia (%)
Canagliflozin	59.1	55.9	63.6
Dapagliflozin	58.9	61.4	46.0
DPP4i	0.81	2.00	2.80
Empagliflozin	81.2	80.7	87.7

### Alzheimer’s disease

**Figure 3:**
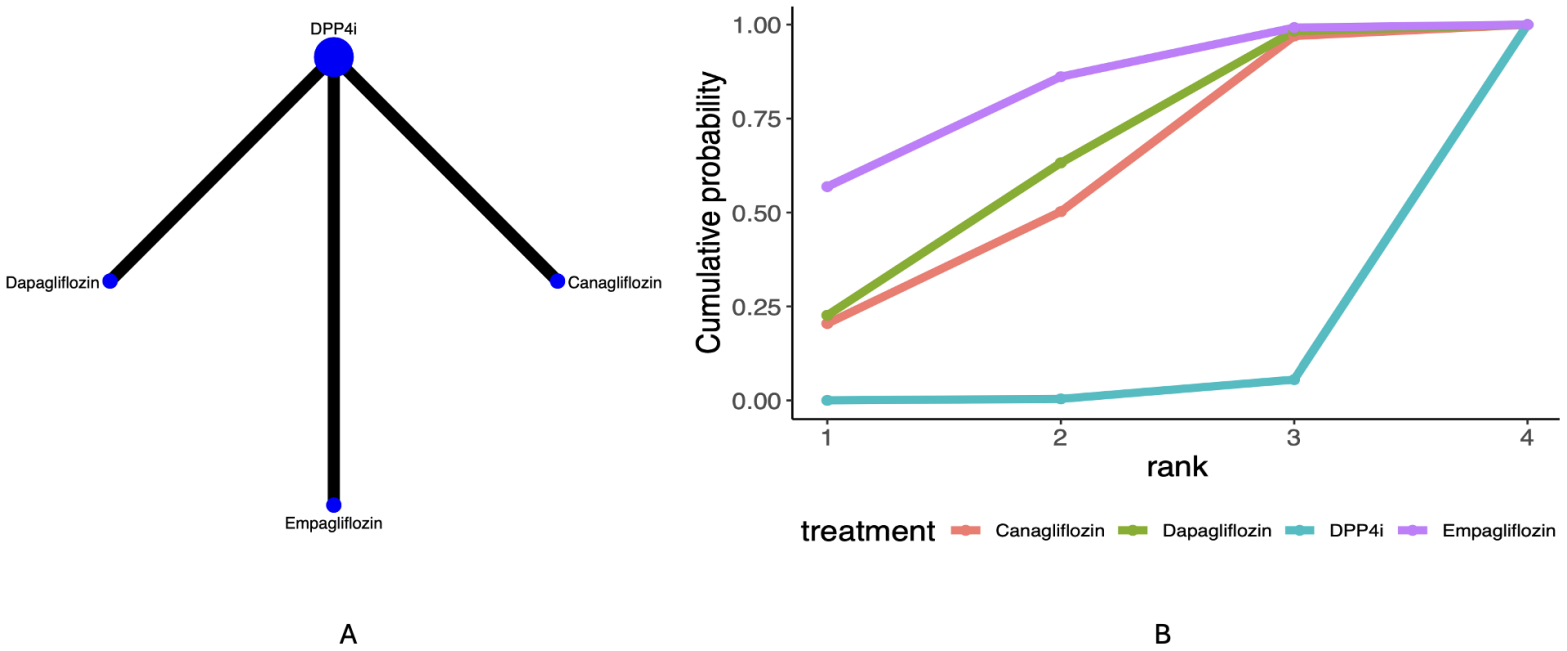
Results of network meta-analysis of Alzheimer’s disease **(A)** network plot; **(B)** cumulative probability plot).

2 studies mentioned Alzheimer’s disease. The network of analyses diagram (**Figure 3A**) indicates direct comparisons between dapagliflozin, empagliflozin, and canagliflozin versus DDP4 inhibitors. The league table (**Supplementary Material** **Supplementary Table 5**) suggests that compared with DDP4 inhibitors, dapagliflozin [HR = 0.68, 95%CrI (0.48, 0.96)], and empagliflozin [HR = 0.62, 95%CrI (0.45, 0.87)] demonstrated reduced Alzheimer’s disease incidence compared to DDP4i. However, empagliflozin showed no statistically significant difference compared to dapagliflozin [HR = 0.91, 95%CrI (0.57, 1.47)] or canagliflozin [HR = 0.89, 95%CrI (0.53, 1.46)]. Cumulative probability curve ranking (**Figure 3B**; **Table 3**) indicated empagliflozin (80.7%) > dapagliflozin (61.4%) > canagliflozin (55.9%).

### Vascular dementia

**Figure 4:**
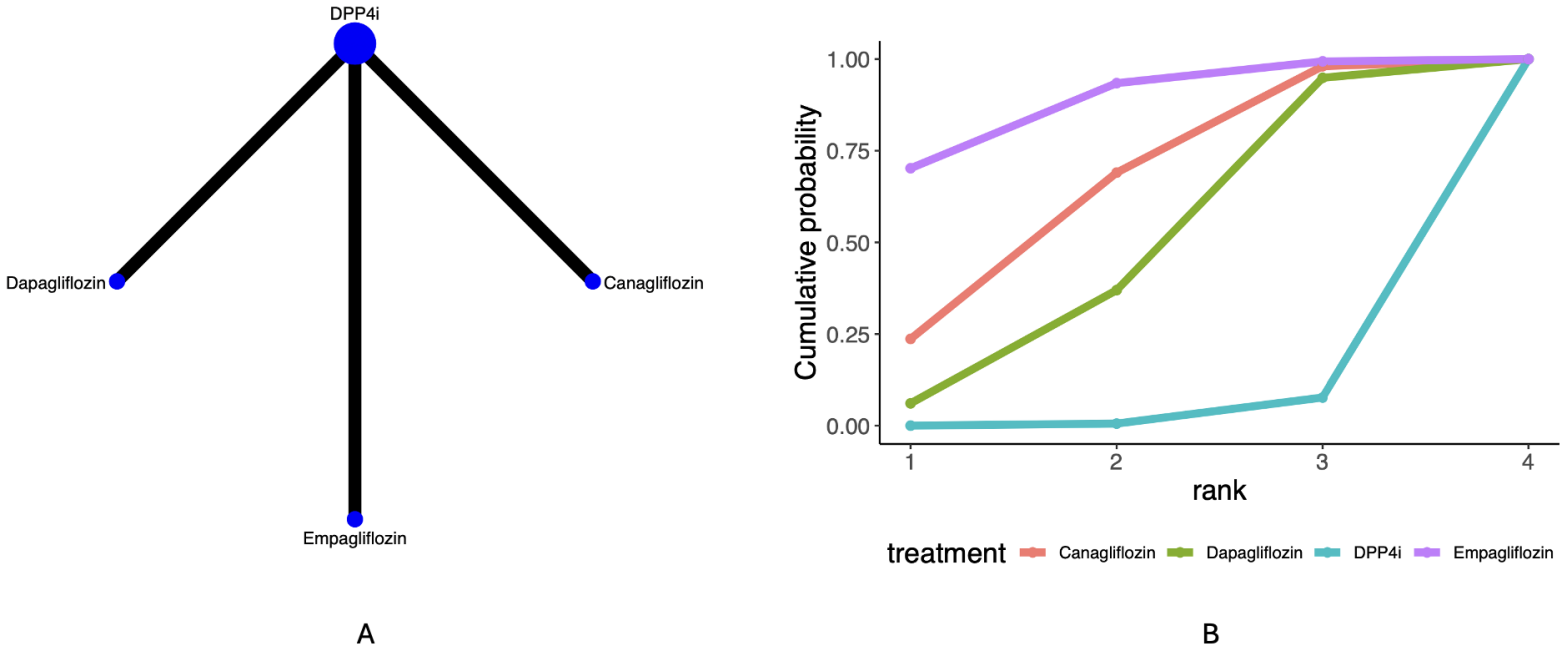
Results of network meta-analysis of vascular dementia **(A)** : network plot; **(B)** cumulative probability plot).

2 studies mentioned Vascular dementia. The network of analyses diagram (**Figure 4A**) indicates direct comparisons between dapagliflozin, empagliflozin, and canagliflozin versus DDP4 inhibitors. The league table (**Supplementary Material** **Supplementary Table 6**) suggests that compared with DDP4 inhibitors, canagliflozin [HR = 0.71, 95%CrI (0.48, 0.98)], and empagliflozin [HR = 0.63, 95%CrI (0.46, 0.87)] demonstrated reduced Vascular dementia incidence compared to DDP4i. However, empagliflozin showed no statistically significant difference compared to dapagliflozin [HR = 0.82, 95%CrI (0.51, 1.24)] or canagliflozin [HR = 0.89, 95%CrI (0.57, 1.49)]. Cumulative probability curve ranking (**Figure 4B**; **Table 3**) indicated empagliflozin (87.7%) > canagliflozin (63.6%) > dapagliflozin (46.0%).

### Publication bias

This study employed the NOS score and funnel plots to detect publication bias. The analysis results (**Supplementary Materials** **Supplementary Figures 1**–**3**) indicate that the funnel plots for dementia, Alzheimer’s disease, or vascular dementia are symmetrical. Furthermore, the Egger test results for dementia (P = 0.125), Alzheimer’s disease (P = 0.17), or vascular dementia (P = 0.445) suggest the presence of publication bias.

## Discussion

This study investigates the impact of different SGLT2 inhibitors on dementia in diabetic patients, representing an innovative aspect compared to previous research. Furthermore, this study aims to evaluate the relationship between various SGLT2i and dementia risk, with a particular focus on comparison with DPP4i. Through a network meta-analysis, Results indicated that compared to DPP4 inhibitors, canagliflozin, dapagliflozin, and empagliflozin demonstrated relative advantages in reducing the incidence of dementia, Alzheimer’s disease, and vascular dementia. Although statistically significant differences were observed between these agents and DPP-4 inhibitors, empagliflozin failed to demonstrate a clear advantage over the other two drugs.

In the dementia outcomes, network analysis revealed that canagliflozin, dapagliflozin, and empagliflozin all significantly reduced dementia incidence compared with DPP-4 inhibitors, with empagliflozin demonstrating particularly marked protective effects at a hazard ratio (HR) of 0.69. This finding aligns with prior research, suggesting SGLT2 inhibitors may further mitigate the risk of brain function decline and cognitive impairment by improving metabolic status in diabetic patients (26). Although empagliflozin had the highest-ranking probability (81.2%), no statistically significant differences were observed between empagliflozin and dapagliflozin or canagliflozin. Therefore, the ranking results should be interpreted cautiously and should not be considered evidence that empagliflozin is superior to the other SGLT2 inhibitors. This noteworthy finding may relate to empagliflozin’s pharmacological properties, its impact on glycemic control, and its potential effects on cerebral blood flow and neuroprotection (27, 28).

Regarding Alzheimer’s disease, analyses revealed that dapagliflozin (HR = 0.68) and empagliflozin (HR = 0.62) demonstrated significant efficacy in reducing disease incidence risk, both outperforming DPP-4 inhibitors. This finding aligns with existing literature, suggesting SGLT2 inhibitors may reduce Alzheimer’s disease incidence in diabetic patients by exerting beneficial effects on cerebral metabolism, inflammatory responses, and neuroprotection. However, no statistically significant difference was observed between empagliflozin and dapagliflozin, suggesting these two agents may exhibit comparable efficacy in preventing Alzheimer’s disease (29, 30). The neurodegenerative processes associated with Alzheimer’s disease appear closely linked to factors such as glycemic control, insulin resistance, oxidative stress, and inflammation. The improvement of these factors may represent the key mechanism through which SGLT2 inhibitors exert their protective effects (31).

Regarding the risk of vascular dementia, results demonstrated that both empagliflozin (HR = 0.63) and canagliflozin (HR = 0.71) exhibited significant efficacy in reducing this risk. Dapagliflozin, however, failed to show statistically significant differences. The development of vascular dementia is closely associated with cerebrovascular events (such as stroke) and vascular health. By controlling blood glucose, SGLT2i may indirectly mitigate vascular lesions, thereby reducing the risk of vascular dementia (32). Although empagliflozin ranked highest in this analysis (87.7%), indicating greater potential for preventing vascular dementia, further clinical data are required to substantiate its efficacy (33). The pathogenesis of vascular dementia is complex, involving factors such as vascular injury, impaired blood-brain barrier function, and oxidative stress. SGLT2 inhibitors may exert their effects through multiple mechanisms (34, 35).

Based on the cumulative probability curve and ranking table, we observed that empagliflozin demonstrated optimal efficacy across all comparisons, exhibiting particularly pronounced relative risk reduction effects for dementia, Alzheimer’s disease, and vascular dementia (81.2%, 80.7%, and 87.7% respectively). This may be attributed to its superior glycemic control, enhanced renal protection, and stronger anti-inflammatory effects. In contrast, dapagliflozin and canagliflozin demonstrated comparable efficacy in reducing dementia risk, though neither was as pronounced as empagliflozin. Notably, while all these agents showed significant effects when compared against DPP-4 inhibitors, the differences between them were not always statistically significant. For instance, direct comparisons between empagliflozin and dapagliflozin or canagliflozin failed to reveal statistically significant differences, suggesting these SGLT2 inhibitors may possess comparable efficacy. In clinical practice, physicians may select appropriate medications based on individual patient circumstances, such as drug tolerance and coexisting conditions.

## Strengths and limitations

The primary strengths of this study lie in its extensive literature search and large sample size, incorporating cohort studies from China, Canada, South Korea, and the United States involving 845,433 patients. This provides robust representativeness and high-quality clinical data. By employing a network meta-analysis approach, we were able to conduct both direct and indirect drug comparisons, thereby comprehensively evaluating the effects of SGLT2 inhibitors such as Dapagliflozin, Empagliflozin, and Canagliflozin on the prevention and treatment of dementia, Alzheimer’s disease, and vascular dementia. Furthermore, this study employed a random-effects model, effectively accounting for heterogeneity across studies and enhancing the robustness and reliability of the analytical results. In the publication bias analysis, neither the funnel plot nor the Egger test indicated significant publication bias, further validating the credibility of the findings.

Despite methodological strengths, this study possesses certain limitations. Firstly, although the included studies featured substantial sample sizes, the overall number of studies remained limited, comprising only six cohort studies. Research specifically addressing Alzheimer’s disease and vascular dementia was particularly scarce, thereby constraining the broad applicability of conclusions. Secondly, baseline differences and inconsistent follow-up durations across studies may have influenced the presentation of drug effects, particularly lacking sufficient data support for long-term efficacy. Furthermore, although a random-effects model was employed, potential confounding factors such as patients’ diabetes control status and other underlying conditions may have influenced the accuracy of the results. Consequently, future large-scale multicenter randomized controlled trials are warranted to validate these findings and further investigate the long-term efficacy and mechanisms of action of SGLT2 inhibitors. In addition, variability in cohort design and study populations should be considered when interpreting our findings. The included studies were conducted in different countries and healthcare systems and were based on different administrative databases or electronic health records. Furthermore, differences in patient characteristics, inclusion criteria, follow-up duration, and baseline risk of dementia may have contributed to between-study heterogeneity, although a random-effects model was used to account for such variability.

## Clinical significance and future prospects

The present study suggests that dapagliflozin, empagliflozin, and canagliflozin are all associated with a lower risk of dementia compared with DPP-4 inhibitors, supporting the potential cognitive benefits of SGLT2 inhibitors in patients with type 2 diabetes. Although empagliflozin had the highest ranking probability in the network meta-analysis, the differences between individual SGLT2 inhibitors were small and no statistically significant differences were observed in the pairwise network comparisons. Therefore, the ranking results should not be interpreted as evidence that one SGLT2 inhibitor is clinically superior to another.

Nevertheless, comparative evidence from network meta-analysis remains clinically valuable because it provides additional information when several SGLT2 inhibitors are appropriate treatment options. In clinical practice, treatment selection should not rely solely on ranking probabilities but should also consider the magnitude and precision of treatment effects, cardiovascular and renal benefits, safety profile, patient comorbidities, drug availability, treatment preferences, and cost. Therefore, the findings of this study may support individualized treatment decisions rather than preferential use of any specific SGLT2 inhibitor.

Future large-scale prospective studies and head-to-head comparative trials are needed to determine whether clinically meaningful differences exist among individual SGLT2 inhibitors with respect to dementia prevention and to further clarify the mechanisms underlying their potential neuroprotective effects.

## Conclusion

The findings suggest that dapagliflozin, empagliflozin, and canagliflozin are all associated with a reduced risk of dementia compared with DPP-4 inhibitors. Although empagliflozin achieved the highest-ranking probability in the network meta-analysis, no statistically significant differences were observed between empagliflozin and the other SGLT2 inhibitors. Therefore, these ranking results should be interpreted with caution. Nevertheless, whilst this study provides encouraging preliminary evidence, further validation of the long-term effects and mechanisms of SGLT2 inhibitors in cognitive function protection is required through additional long-term, multicenter clinical trials.

## Data Availability

The original contributions presented in the study are included in the article/**Supplementary Material**. Further inquiries can be directed to the corresponding author.
