# Supplementary material for: Association between different sodium–glucose co-transporter 2 inhibitors and the risk of dementia: a network meta-analysis
Source: Front Endocrinol (Lausanne). 2026 Aug 4;17:1777151. doi: 10.3389/fendo.2026.1777151 (PMC13481255; doi:10.3389/fendo.2026.1777151)
Supplement: Supplementary file 1 [file DataSheet1.docx]

Table S1 search strategy

(("Sodium-Glucose Transporter 2 Inhibitors"[Mesh]) OR ((((((((((((((((((Sodium-Glucose Transporter 2 Inhibitors[Title/Abstract]) OR (Sodium Glucose Transporter 2 Inhibitors[Title/Abstract])) OR (SGLT-2 Inhibitors[Title/Abstract])) OR (SGLT 2 Inhibitors[Title/Abstract])) OR (SGLT-2 Inhibitor[Title/Abstract])) OR (Inhibitor, SGLT-2[Title/Abstract])) OR (SGLT 2 Inhibitor[Title/Abstract])) OR (Sodium-Glucose Transporter 2 Inhibitor[Title/Abstract])) OR (Sodium Glucose Transporter 2 Inhibitor[Title/Abstract])) OR (SGLT2 Inhibitor[Title/Abstract])) OR (Inhibitor, SGLT2[Title/Abstract])) OR (Gliflozins[Title/Abstract])) OR (Gliflozin[Title/Abstract])) OR (SGLT2 Inhibitors[Title/Abstract])) OR (Empagliflozin[Title/Abstract])) OR (Dapagliflozin[Title/Abstract])) OR (Canagliflozin[Title/Abstract])) OR (Ertugliflozin[Title/Abstract]))) AND (("Dementia"[Mesh]) OR (((((((((((((((((((((((((Dementia[Title/Abstract]) OR (Dementias[Title/Abstract])) OR (Amentia[Title/Abstract])) OR (Amentias[Title/Abstract])) OR (Senile Paranoid Dementia[Title/Abstract])) OR (Dementias, Senile Paranoid[Title/Abstract])) OR (Paranoid Dementia, Senile[Title/Abstract])) OR (Paranoid Dementias, Senile[Title/Abstract])) OR (Senile Paranoid Dementias[Title/Abstract])) OR (Familial Dementia[Title/Abstract])) OR (Dementia, Familial[Title/Abstract])) OR (Dementias, Familial[Title/Abstract])) OR (Familial Dementias[Title/Abstract])) OR (Vascular dementia[Title/Abstract])) OR (Dementias, Vascular[Title/Abstract])) OR (Vascular Dementias[Title/Abstract])) OR (Vascular Dementia[Title/Abstract])) OR (Arteriosclerotic Dementia[Title/Abstract])) OR (Arteriosclerotic Dementias[Title/Abstract])) OR (Dementia, Arteriosclerotic[Title/Abstract])) OR (Dementias, Arteriosclerotic[Title/Abstract])) OR (Binswanger Disease[Title/Abstract])) OR (Disease, Binswanger[Title/Abstract])) OR (Alzheimer's disease[Title/Abstract])) OR (Alzheimer Syndrome[Title/Abstract])))

Table S2 NOS results

| cohort study | | | | | | | | | |
| --- | --- | --- | --- | --- | --- | --- | --- | --- | --- |
| Study | Representativeness of the exposed group | Selection of non-exposed groups | Determination of exposure factors | Identification of outcome indicators not yet to be observed at study entry | Comparability of exposed and unexposed groups considered in design and statistical analysis | design and statistical analysis | Adequacy of the study's evaluation of the outcome | Adequacy of follow-up in exposed and unexposed groups | Total scores |
| Abdullah2025 | * | * | * | * | ** | * | * | * | 9 |
| Chiang2025 | * | * | * | * | ** | * | * | * | 9 |
| Hong2024 | * | * | * | / | ** | * | * | * | 8 |
| Liu2025 | * | * | * | * | ** | * | * | * | 9 |
| Tang2025 | * | * | * | * | * | * | * | * | 8 |
| Wu2025 | * | * | * | * | * | * | * | * | 8 |

Table S3 Results of consistency modeling

| Outcome | DIC (Consistency model) | DIC (Inconsistency model) | ΔDIC | Between-study heterogeneity (τ) | Interpretation |
| --- | --- | --- | --- | --- | --- |
| Dementia | 16.42 | 17.01 | 0.59 | 0.08 | Low heterogeneity |
| Alzheimer's disease | 8.57 | 9.23 | 0.66 | 0.1 | Low heterogeneity |
| Vascular dementia | 7.94 | 8.41 | 0.47 | 0.11 | Low heterogeneity |

Table S4 Dementia League Table

| HR 95%CrI | | | |
| --- | --- | --- | --- |
| Canagliflozin |  |  | 0.92 (0.65, 1.26) |
| 1 (0.72, 1.41) | Dapagliflozin | 1.33 (1.07, 1.66)* | 0.92 (0.67, 1.24) |
| 0.75 (0.59, 0.98)* | 0.75 (0.6, 0.93)* | DPP4i | 0.69 (0.55, 0.85)* |
| 1.09 (0.79, 1.54) | 1.09 (0.81, 1.48) | 1.45 (1.18, 1.81)* | Empagliflozin |

* means P<0.05

Table S5 Alzheimer’s disease League Table

| HR 95%CrI | | | |
| --- | --- | --- | --- |
| Canagliflozin |  |  | 0.89 (0.53, 1.46) |
| 1.03 (0.62, 1.72) | Dapagliflozin | 1.46 (1.04, 2.07) * | 0.91 (0.57, 1.47) |
| 0.7 (0.48, 1.03) | 0.68 (0.48, 0.96) * | DPP4i | 0.62 (0.45, 0.87) * |
| 1.13 (0.68, 1.88) | 1.09 (0.68, 1.77) | 1.6 (1.14, 2.25) | Empagliflozin |

Table S6 Vascular dementia League Table

| HR 95%CrI | | | |
| --- | --- | --- | --- |
| Canagliflozin |  |  | 0.89 (0.57, 1.49) |
| 0.92 (0.53, 1.39) | Dapagliflozin | 1.3 (0.91, 1.72) | 0.82 (0.51, 1.24) |
| 0.71 (0.48, 0.98)* | 0.77 (0.58, 1.1) | DPP4i | 0.63 (0.46, 0.87)* |
| 1.13 (0.67, 1.75) | 1.21 (0.81, 1.97) | 1.58 (1.15, 2.16) | Empagliflozin |

Figure S1 Meta-analysis funnel plot of dementia.

Figure S2 Meta-analysis funnel plot of Alzheimer's disease.

Figure S3 Meta-analysis funnel plot of vascular dementia.

Figure S4. Gelman–Rubin convergence diagnostics for the Bayesian network meta-analysis.
